# New concepts for glioblastoma vaccine immunotherapy

**DOI:** 10.1016/j.bj.2026.100974

**Published:** 2026-03-28

**Authors:** Mariam Shallak, Amruth K.B. Shaik, Andrea Gatta, Mattia Volpi, Roberto S. Accolla, Greta Forlani

**Affiliations:** a Department of Medicine and Technological Innovation, University of Insubria, 21100, Varese, Italy; b Department of Biotechnology and Biosciences, University of Milano-Bicocca, Milano, Italy; c Cellular and Molecular Oncoimmunology Lab, IRCCS Humanitas Research Hospital, Milan, Italy

**Keywords:** Glioblastoma, Immunovirology, Immuno-oncology, Peptide-vaccine, CIITA, MHC-II, Oncolytic viruses

## Abstract

**Background:**

Glioblastoma remains one of the most lethal malignancies, characterized by rapid recurrence, profound intratumoral heterogeneity, and a highly immunosuppressive microenvironment.

Among immunotherapeutic strategies, peptide-based vaccines have attracted attention for their safety, specificity, and capacity to elicit tumor-directed T cell responses. Over the past two decades, several platforms targeting tumor-associated or tumor-specific antigens, including EGFRvIII, WT1, and survivin, have advanced into clinical trials. While early-phase studies demonstrated immunogenicity and occasional survival benefits, phase III trials have largely failed to confirm durable efficacy, underscoring the challenges posed by the ongoing complexity of tumor evasion mechanisms among which down regulation of MHC (HLA) expression in tumor cells, lack or reduced tumor antigen expression and a suppressive tumor microenvironment certainly play a role.

**Main body:**

As far as tumor antigens, recent insights also question the centrality of neoantigens, highlighting instead the immunogenicity of shared tumor-associated antigens, which may provide a more reliable foundation for broadly applicable vaccines in GBM. A major barrier to efficacy remains impaired antigen presentation, particularly the downregulation of MHC-II pathways. In this context, strategies leveraging the transcriptional activator CIITA to restore MHC-II expression hold promise both for reprogramming GBM cells into effective antigen-presenting cells and for the isolation of new families of MHC class II-bound peptides relevant for the triggering of tumor-specific CD4^+^ T cells.

**Conclusion:**

This new approach could pave the way for next-generation peptide vaccines, particularly when integrated with combinatorial modalities such as checkpoint inhibitors, myeloid-targeted therapies, or oncolytic viruses.

## Highlights

• Peptide vaccines for glioblastoma are immunogenic but clinically limited.
• Immune evasion and poor antigen presentation restrict vaccine efficacy.
• Restoring MHC II expression in tumor cells may enhance antitumor immunity.

## 1 Introduction

Glioblastoma (GBM) is the most aggressive primary brain tumor in adults, accounting for approximately 80% of all malignant brain tumors [1]. The current standard of care includes maximal surgical resection followed by radiotherapy and adjuvant chemotherapy with temozolomide [2]. Despite this multimodal approach, the median overall survival is approximately 15 months [3,4]. Standard therapies not only fail to eradicate GBM but also impose selective pressures that result in the appearance of resistant clones, pronounced genetic variability and extensive intratumoral heterogeneity [[5], [6], [7], [8]].

The therapeutic challenge posed by GBM is further worsened by the anatomical location of the tumor and by the presence of the blood-brain barrier (BBB), limiting the delivery of therapeutic compounds to the tumor site [9]. Moreover, GBM is characterized by a highly immunosuppressive tumor microenvironment (TME), which facilitates tumor progression [10]. Indeed, GBM cells actively recruit immunosuppressive myeloid-derived suppressor cells (MDSC), tumor-associated macrophages (TAMs), and regulatory T lymphocytes (Tregs), creating a niche that inhibits effective anti-tumor immune responses [11]. This underscores the urgent need for novel therapeutic strategies that could improve patient outcomes.

In recent years, the introduction of immunotherapeutic approaches, particularly the use of immune checkpoint inhibitors (ICI) has revolutionized the strategy of anti-tumor therapies, resulting in unprecedented success in treatment of certain tumors, such as melanoma and non-small cell lung cancer. However, translating these successes to GBM has proven exceptionally challenging. The poor outcome of ICI in GBM finds is largely attributable to the profoundly immunosuppressive tumor microenvironment. Within this milieu, several cellular populations, including Tregs, MDSC and TAM, as discussed in our review, play major roles, both in limiting the infiltration of anti-tumor T cells (including both T helper and CTL) as well as in suppressing their function [12]. The rather poor success of ICI in GBM was in part justified by the prevalent belief that the brain is an "immune-privileged" organ because of the BBB [13]. The concept of brain as immune privileged organ remained largely unchallenged until recently when it was discovered the existence of a central nervous system (CNS) lymphatic system within the meninges [14,15] as well as a local source of functional immune cells residing in the bone marrow of the skull that can be mobilized into the brain via cerebrospinal fluid (CSF) [16,17]. These findings have reinvigorated the rationale for immunotherapy in GBM. Moreover, recent advances in nanomedicine have enabled the development of nanoscale delivery systems aimed to better cross the BBB and enhance tumor targeting [12,[18], [19], [20]]. However, the ability of nanoparticles to effectively cross the BBB remains limited and depends on several factors, including ultrasound-mediated BBB disruption, access to GBM vasculature, the enhanced permeability and retention (EPR) effect, tumor heterogeneity and the optimization of targeting moieties or ligands used to modify nanosystems. In this review we will first summarize the evidence that make GBM particularly reluctant to therapies and then describe recent immunotherapeutic vaccination approaches among which one, based on the rescue of Major Histocompatibility Complex class II (MHC-II) gene expression in tumor cells, is in our opinion a very promising and innovative way to allow tumor cells themselves to become surrogate APCs with the consequent display of an unprecedented repertoire of tumor antigens for tumor-specific T cell scrutiny.

## 2 The immunosuppressive tumor microenvironment in GBM

The immune microenvironment of GBM is a highly complex and dynamic system that critically modulates the effectiveness of immunotherapeutic interventions. GBM orchestrates a profoundly immunosuppressive milieu, enabling immune evasion and presenting a substantial barrier to the development of effective therapeutic strategies. GBM tumors actively recruit Tregs, MDSCs, and TAMs which secrete immunosuppressive cytokines like TGF-β, IL-10, and prostaglandin E2 (PGE2), all of which collectively dampen anti-tumor immunity [21].

### 2.1 Regulatory T cells (Tregs)

Tregs contribute to immune evasion not only by promoting immunosuppressive cytokine production but also by directly attenuating anti-tumor immune responses [22]. Treg-mediated immunosuppression is partially associated to the expression of heme oxygenase-1 (HO-1), which has been shown to suppress T-cell proliferation and reduce sensitivity to apoptosis through inhibition of death receptor expression, thereby enhancing immune tolerance to tumor cells [23]. Moreover, Tregs contribute to tumor progression by promoting glioma stemness via the TGF-β–NF–κB-IL-6-STAT3 signalling axis, thereby enhancing tumor aggressiveness [24,25]. Clinical and experimental data also indicate a positive correlation between glioma grade and Tregs infiltration. Glioblastomas (grade IV gliomas) exhibit the highest levels of FOXP3 expression and significantly elevated HO-1 mRNA levels in CD4^+^CD25^+^ Tregs compared to lower-grade gliomas, highlighting a potential link between HO-1 activity and FOXP3-mediated immunosuppression during GBM progression [26]. As recently demonstrated by Salahlou and colleagues, Tregs further suppress the function of antigen-presenting cells (APCs) such as dendritic cells, and effector lymphocytes by upregulating immunosuppressive mediators such as IL-10, TGF-β, and IDO, thereby reinforcing an immunosuppressive tumor milieu and posing a major obstacle to effective immunotherapy [27].

### 2.2 Myeloid-derived suppressor cells (MDSCs)

Importantly, the expansion and activation of Tregs in the GBM microenvironment are closely supported by MDSCs, which secrete high levels of immunosuppressive cytokines that not only promote Tregs induction but also inhibit cytotoxic immune responses. They produce various chemokines and growth factors, such as CCL3, CCL4, and CCL5, that actively recruit Tregs into the tumor microenvironment, further intensifying immunosuppression. Moreover, MDSCs release elevated levels of immunosuppressive cytokines and enzymes, including TNF-α, TGF-β, IL-10, IL-12, and indoleamine 2,3-dioxygenase, which collectively inhibit anti-tumor immune responses and contribute to resistance against immunotherapeutic interventions [28,29]. In addition to their immunoregulatory functions, MDSCs contribute to GBM progression by promoting cancer stem cell phenotypes and facilitating epithelial-mesenchymal transition, thereby enhancing tumor invasiveness and dissemination. The expansion and recruitment of MDSCs into the tumor microenvironment is fostered by tumor-derived cytokines, including IL-6, IL-8, IL-10, Colony Stimulating Factor 1 (CSF-1), CCL2, CXCL2, PGE2, and TGF-β. Additionally, hypoxic conditions within the tumor niche reprogram MDSCs metabolism toward fatty acid oxidation, thereby enhancing their immunosuppressive capacity [29,30].

### 2.3 Tumor-associated macrophages (TAMs)

TAMs constitute a significant proportion of the GBM TME and are predominantly polarized into pro-tumorigenic M2-like macrophages that promote angiogenesis, suppress T cell responses, thus favouring immune evasion and tumor progression [31]. The primary TAM populations identified in GBM include resident CNS microglia and infiltrating bone marrow-derived monocytes. TAMs exhibit reduced expression of co-stimulatory molecules critical for lymphocyte activation, including CD40, CD80, and CD86, thereby limiting T cell priming [32]. Instead, these cells express high levels of immunosuppressive ligands such as Programmed Death Ligand 1 (PD-L1**)** and Fas ligand (FasL), further contributing to T cell exhaustion and apoptosis [33]. Additionally, in the contest of GBM, the MHC–II–dependent antigen presentation of TAMs is impaired [34].

### 2.4 Loss or downregulation of MHC expression

The immunosuppressive GBM microenvironment not only inhibits immune cell infiltration and function but also shapes tumor-intrinsic mechanisms that facilitate immune escape. One of the most prominent and clinically relevant of these mechanisms is the downregulation of MHC molecules which severely limits antigen presentation and T cell-mediated tumor recognition.

Indeed, the downregulation of MHC molecules is a hallmark of GBM's immune evasion strategy. Analysis of MHC-I antigens in GBM patients demonstrated that nearly 50% of cases exhibited a loss of HLA-A2 expression. Moreover, MHC-I antigen expression showed a positive correlation with tumor grade [35]. One study reported concurrent downregulation of co-stimulatory molecules alongside MHC-I antigen expression, both of which were directly associated with glioma grade [36]*.* Moreover, mutations in the antigen presentation machinery, such as TAP1/2, or Tapasin result in marked decrease in peptide-loaded MHC-I expression to the cell surface [10,37,38]. In addition, it was reported that the long non-coding RNA LINC01232 is upregulated and increases the expression of the transcription factor E2F2. E2F2 enhances NBR1 expression, which binds to MHC-I and mediates its ubiquitination and degradation through the autophagy-lysosome system [39].

Besides the well-characterized downregulation of MHC-I molecules in GBM, MHC-II expression also appears to be frequently impaired, further dampening anti-tumor immune surveillance. In glioblastoma, alterations in the biosynthetic machinery necessary for MHC-II expression, particularly at the level of transcriptional regulation, may contribute to this deficiency [32].

The widespread absence of MHC-II expression prevents efficient CD4^+^ T cell priming and activation. This is particularly detrimental, since CD4^+^ T cells provide essential “helper” support for CD8^+^ T cells, sustaining their expansion, function, and memory. Thus, impaired MHC-II expression not only reduces CD4^+^ effector responses but also compromises CTL activity. Importantly, defects in interferon-γ (IFN-γ) signalling have also been reported in GBM, including alterations in JAK/STAT components [40]. As IFN-γ is a master cytokine for upregulation of MHC class I and, most importantly, MHC-II expression, it derives that these changes blunt tumor ability to be recognized by T cells, further enforcing immune escape and limiting the efficacy of therapies that depend on IFN-γ-mediated antigen presentation. The central role of IFN-γ in controlling MHC-II expression converges on the regulation of CIITA, a non-DNA-binding coactivator that serves as the master transcriptional regulator of MHC-II genes [[41], [42], [43], [44], [45], [46]]. Thus, defects in IFN-γ signalling globally affect tumor antigen presentation and tumor specific T cell activation.

## 3 Present vaccination approaches in GBM

### 3.1 Peptide-based vaccines in GBM: advantages and challenges

Cancer vaccines are designed to stimulate the adaptive immune system against tumor antigens and have emerged as an area of active investigation in GBM.

Tumor antigens can be broadly divided into tumor-specific antigens (TSAs), also referred to as neoantigens, which arise from somatic mutations, and tumor-associated antigens (TAAs), which are non-mutated proteins aberrantly expressed in tumor cells but largely absent in normal tissues [47]. TSAs are theoretically ideal targets due to their tumor exclusivity, yet their frequency in GBM is limited given its relatively low mutational burden. By contrast, TAAs such as epidermal growth factor receptor (EGFR**)**, survivin, and Wilms tumor 1 (WT1**)** are more consistently expressed across patients, making them attractive targets for vaccine development. In addition, exogenous antigens derived from viral infections, most notably cytomegalovirus (CMV), have been identified in a subset of GBM tumors, offering another potential immunogenic source [48]. Vaccination strategies may exploit predefined antigens (shared antigens expressed in multiple patients or personalized antigens tailored to an individual's tumor profile) or utilize “anonymous” approaches, where complex antigen mixtures such as tumor lysates or nucleic acids are presented by APCs. Upon vaccination, APCs process and display antigenic peptides via MHC molecules to T cells, initiating cytotoxic responses against tumor cells and ideally generating long-term memory to prevent recurrence. Due to their inability to intrinsically activate pattern recognition receptors or other innate immune pathways, peptide-based vaccines are typically formulated with immune adjuvants that provide the requisite costimulatory signals and cytokine milieu to APCs, thereby facilitating efficient priming and expansion of antigen-specific CD4^+^ and CD8^+^ T cells [49]. Several vaccine platforms have been explored in GBM, including peptide vaccines, dendritic cell (DC) vaccines, nucleic acid-based vaccines, and viral vector vaccines. Among these, peptide-based approaches have been most extensively studied, owing to their precision of antigen targeting, relative simplicity and safety of manufacturing, and the possibility of combining multiple epitopes to broaden immune coverage [27]. Preclinical models also suggest that engineered peptide variants with optimized processing can synergize with checkpoint blockade, enhancing therapeutic efficacy [50].

Over the past two decades, several clinical and preclinical studies have evaluated peptide-based vaccines in GBM. In [Table 1] the principal clinical trials are described. Peptide vaccines utilize synthetic peptides to mimic tumor-associated or tumor-specific epitopes, aiming to elicit primarily cytotoxic T cell responses. Rindopepimut (CDX-110) is one of the most extensively studied peptide vaccines, targeting the EGFRvIII, a mutation found in a substantial proportion of GBM cases. Early Phase II trials reported robust immune activation and prolonged survival [51,52]. However, the Phase III ACT IV trial failed to demonstrate overall survival benefit in newly diagnosed patients. Subsequent analyses indicated that antigen loss and intratumoral heterogeneity contributed to the lack of durable benefit, highlighting a major challenge for predefined peptide-based vaccines [53].

**Table 1:** Most relevant clinical Trials of Peptide Vaccines in Glioblastoma.

Vaccine	Target Antigen	Trial Phase	Patient Population	Key Outcomes	Limitations
Rindopepimut (CDX-110) [[51], [52], [53]]	EGFRvIII	Phase II → Phase III (ACT IV, NCT01480479)	Newly diagnosed GBM	Phase II: immune activation and prolonged PFS; Phase III: no OS benefit (mOS 20.1 vs. 20.0 mo)	Antigen loss, intratumoral heterogeneity
DSP-7888 [54]	WT1	Phase I–III (NCT03149003)	Recurrent/progressive GBM	Safe, well tolerated; Phase III with bevacizumab: no OS improvement	High WT1 expression required, modest efficacy
SurVaxM [55]	Survivin	Phase I–II (NCT02455557)	Newly diagnosed GBM	mPFS 11.4 mo, mOS 25.9 mo vs. historical controls	Small cohorts, ongoing phase IIb
IMA950 [58]	Multi-peptide (shared TAAs)	Phase I/II (NCT01920191)	Newly diagnosed GBM	PFS rates were 74% at 6 mo and 31% at 9 mo. Induced multi-epitope T cell responses	No OS improvement
HSPPC-96 [59]	TSA	Phase II (NCT00293423)	Recurrent GBM	mOS 9.9 mo	No OS improvement
Personalized vaccine (GAPVAC-101) [60]	TSA	Phase I (NCT02149225)	Newly diagnosed GBM	mPFS 14.2 mo, mOS 29 mo. Feasible, immunogenic, induced CD4/CD8 memory	Limited efficacy, variability in neoantigen expression
Personalized vaccine [61]	TSA	Phase I (NCT02287428)	Newly diagnosed personalized vaccines MGMT unmethylated	mPFS 7.6 mo, mOS 16.8 mo. Feasible, immunogenic, induced CD4/CD8 memory	Limited efficacy, variability in neoantigen expression

Median progression-free survival (mPSF); Median overall survival (mOS); months (mo). References are indicated by numbers in square brackets.

Similar findings were observed with peptide vaccines targeting WT1 [Table 1]. The DSP-788 vaccine, consisting of synthetic peptides derived from WT1, showed good tolerability in early-phase studies. A Phase II trial demonstrated safety and disease stabilization in recurrent GBM [54]. However, a subsequent Phase III trial combining DSP-788 with bevacizumab did not improve clinical outcomes compared with bevacizumab alone (ClinicalTri-als.gov identifier: NCT03149003; https://clinicaltrials.gov/study/NCT03149003). Others peptide vaccines, such as SurVaxM (targeting survivin), IMA950 (a multipeptide platform), and the HSPPC-96 peptide complex, have shown safety and immunogenicity in early-phase trials [[55], [56], [57], [58]].

The negative results of clinical trials with Rindopepimut and DSP-7888 underscore several critical challenges: the rapid loss of target antigen under immune pressure (antigen escape), the profound intratumoral heterogeneity of GBM that limits the durability of single-antigen approaches, and the constraints of MHC restriction that reduce applicability across patients. Beside antigen loss, several additional factors may explain the limited success of current peptide vaccines in phase III clinical trials. Many vaccine formulations rely on a single or limited number of tumor-specific or tumor-associated epitopes that prevents the stimulation of a large repertoire of tumor-reactive lymphocytes. Furthermore, and of relevant importance, many peptide vaccine strategies do not adequately stimulate anti-tumor CD4^+^ T helper cells. Vaccine formulations are often focused on peptides recognized by the terminal effectors CTL. However, CTLs cannot sustain long-term proliferation and functional persistence in the absence of adequate CD4^+^ T helper cell support.

Collectively, these lessons emphasize the necessity of multi-antigen vaccines and strategies to stabilize antigen presentation in order to achieve meaningful clinical impact.

Personalized peptide vaccination strategies have also been developed, selecting epitopes based on patient MHC haplotype and tumor antigen profile. A Phase I trial in HLA-A24+ patients demonstrated feasibility and antigen-specific immune responses [59]. With the advent of next generation sequencing, neoantigen vaccines have further expanded this approach. Proof-of-concept studies demonstrated induction of both CD4^+^ and CD8^+^ T cell responses, with some peptides recognized by intratumoral lymphocytes and mediating tumor cell lysis [60,61].

Recent large-scale analyses by Apavaloaei et al. have challenged the long-held assumption that mutated neoantigens represent the dominant actionable antigens in cancer. Their findings that only ∼1% of tumor antigens are mutated TSAs (mTSAs), while the vast majority are shared (TAAs) or aberrantly expressed TAAs (aeTSAs), or lineage-specific antigens (LSAs) [62], sign a paradigm shift that if extrapolated to GBM, could be particularly relevant as this tumor is characterized by an inherently low mutational burden. Together, these lessons highlight the limitations of relying on narrow, individualized neoantigen-based approaches and instead point toward the possibility of targeting shared TAAs and physiologically presented peptides as the foundation for next-generation vaccine design. However, it should be noted that shared antigens do not necessarily guarantee safe or more effective therapeutic outcomes, as many TAAs are subject to immune tolerance and may also be expressed in normal tissues, thereby posing potential off-target risks.

### 3.2 Dendritic cell vaccines (DC vaccines)

DCs are professional APC capable of priming both CD4^+^ and CD8^+^ T cells, making them attractive for vaccine development. DC vaccines are generated by harvesting autologous DCs from patients, loading them *ex vivo* with tumor antigens (peptides, RNA/DNA, or tumor lysates), and reinfusing them to induce antitumor immunity [63]. In GBM, ICT-107 is a well-studied autologous DC vaccine pulsed with six synthetic peptides restricted to HLA-A1 and HLA-A2 alleles [Table 2]. In a randomized Phase II trial, ICT-107 was well tolerated and associated with a modest but significant improvement in progression-free survival, although overall survival benefit did not reach statistical significance in the intent-to-treat population [64,65].

**Table 2:** Most relevant clinical Trials of Dendritic Cell Vaccines in Glioblastoma.

Vaccine	Antigen Source/Target	Trial Phase	Patient Population	Key Outcomes	Limitations
ICT-107 [65]	Six synthetic peptides (HLA-A1/A2 restricted: AIM2, MAGE1, TRP-2, gp100, HER2, IL13Rα2)	Phase II (NCT01280552)	Newly diagnosed GBM	Median OS 18.3 mo vs. 16.7 mo in control; PFS benefit in HLA-A2+ subgroup	Benefit limited to subgroups, small sample size, lack of phase III confirmation
DCVax-L [66,67]	Autologous tumor lysate	Phase III (NCT00045968)	Newly diagnosed GBM and recurrent GBM	Median OS 19.3 vs. 16.5 mo in newly diagnosed. 13.2 vs. 7.8 mo in recurrent GBM. ∼23.1 mo vs. ∼15-17 mo in external controls; long-term survivors observed	No randomized control arm; external control group introduces bias
CMV-DC [68]	CMV-pp65	Phase II (NCT00639639)	Newly diagnosed GBM	Median OS ∼37.7-38.3 mo vs. ∼14.6-20.9 mo, in controls receiving standard of care and adjuvant therapy. Safe, immunogenic; Induction a CMV-specifc CD8^+^ T-cell response	Improve survival compared to standard of care

Median progression-free survival (mPSF); Median overall survival (mOS); months (mo). References are indicated by numbers in square brackets.

The most advanced DC vaccine is DCVax-L, an autologous lysate-pulsed DC product. In a large Phase III trial (NCT00045968), DCVax-L treatment was associated with limited improved overall survival compared with external controls, both in newly diagnosed (19.3 vs. 16.5 months) and recurrent GBM (13.2 vs. 7.8 months) patients. However, interpretation is complicated by crossover design and methodological limitations [66,67]. Other platforms, such as CMV-DCs, have also been tested, leveraging the presence of viral antigens in GBM to induce strong T cell responses, though these remain in early-phase trials [68] [Table 2].

In consideration of the above results, it appears that present DC-based strategies face a series of limitations ranging from the use of a single or poorly immunogenic antigens, the fact that the peptides loaded onto DCs are typically predicted through computational algorithms and may not accurately reflect the repertoire of antigens naturally processed and presented by tumor cells, the choice of the optimal route of administration that may greatly affect DC vaccine efficacy [69].

Whatever the reason, the efficacy of DC vaccination will ultimately be dependent on efficient antigen presentation. Given the frequent downregulation of MHC-II in GBM, and the emerging role of CIITA in restoring this pathway, combinatorial strategies that couple peptide vaccination with CIITA-mediated MHC-II induction may provide a way to overcome current limitations (see below).

### 3.3 Changing the paradigm: using CIITA to make GBM cells APCs of their own tumor antigens

As mentioned above, CIITA is the major regulator of MHC-II gene expression and its own expression can be induced in classical APCs by IFN-γ through two distinct CIITA promoters designated pIII and pIV.

Interestingly, GBM cells have been shown to upregulate CIITA upon IFN-γ stimulation, utilizing both pIV and pIII promoters. This dual promoter usage suggests a degree of transcriptional plasticity in glioma cells, enabling them to potentially acquire APC-like functions under inflammatory conditions. Indeed, *in vitro* and *in vivo* analyses demonstrated that GBM cells can process and present native antigens to CD4^+^ MHC–II–restricted Th1 cells. This capability implies that, when appropriately stimulated, GBM cells may not be entirely invisible to CD4^+^ T cell surveillance, though this potential is often suppressed in the native tumor microenvironment [70,71]. Moreover, emerging transcriptomic analyses support the existence of GBM subpopulations with elevated expression of APC-related genes, including HLA-DRA, CD74, CD80, CD86, and CIITA. These APC-high states correlate with reduced stemness signatures, enhanced inflammatory signalling, and a distinct metabolic reprogramming profile characterized by decreased oxidative phosphorylation and increased lipid/steroid metabolism. These features may reflect a more differentiated tumor cell phenotype, which is also more immunogenically "visible" and potentially responsive to immune-based therapies [72]. Elevated CIITA expression and MHC-II activity in these subpopulations could serve as biomarkers for increased CD4^+^ T cell engagement and better therapeutic responsiveness, particularly in the context of peptide-based vaccination strategies that rely on robust TH cell activation. Experimental studies in animal models have demonstrated the potential of CIITA-based approaches to render tumor cells immunogenic in vivo and to reprogram the immune landscape within the tumor microenvironment. Notably, CIITA expression enables tumor cells to function as surrogate antigen-presenting cells, capable of priming naïve, tumor-specific CD4^+^ T cells [[73], [74], [75], [76]]. In glioblastoma specifically, CIITA-transfected GBM cells have shown delayed growth or complete rejection *in vivo*, associated with increased infiltration of both CD4^+^ and CD8^+^ T lymphocytes into the tumor bed [74,77]. These effects suggest an important role of CIITA in breaking immune tolerance in the GBM microenvironment. We emphasize that, in our experience with numerous tumor animal models including both carcinomas and sarcomas, we have never observed pathological signs of autoimmunity in animals injected with CIITA-modified tumor cells, even after prolonged observation periods (for a review see Ref. [44]). Similarly, the induction of T cell anergy was not detected. This is supported by the observation that animals rejecting CIITA-expressing tumors remained resistant when subsequently challenged with parental tumor cells. More importantly, spleen cells isolated from these immune animals were able to transfer long-lasting protection against the parental tumor when injected into naïve recipients. Therefore, although potential risks cannot be entirely excluded when translating this strategy to the clinical setting, our findings provide strong evidence that this approach has a solid safety basis and merits careful consideration for therapeutic application. One particularly important outcome of these strategies has been the identification of a broad repertoire of tumor-specific, immunogenic peptides presented on MHC-II molecules, which may serve as candidate components for personalized tumor vaccines [78]. Notably, our group has recently shown that vaccination with CIITA-modified GBM cells protects mice not only against rechallenge with the same tumor, but also against a different, unmodified GBM tumor line. This cross-protection implies the presence of shared immunogenic peptides between distinct GBM models, supporting the concept that CIITA-based strategies may uncover relevant tumor antigens across patients [79]. Altogether, these findings support the therapeutic rationale for modulating CIITA expression and restoring MHC-II functionality in GBM. By reprogramming GBM cells into surrogate professional APCs, through CIITA gene delivery may thus represent a novel strategy to facilitate CD4^+^ T cell priming, and ultimately enhance the efficacy of peptide-based immunotherapy. Beyond facilitating the discovery of physiologically relevant MHC-II restricted peptides for novel vaccine formulations, CIITA-mediated MHC-II expression in tumor cells provides a means to enhance the tumor's immunogenicity and to potentially synergize with other immune-based treatment strategies such as checkpoint blockade or oncolytic virotherapy.

## 4 Conclusions

**Fig. 1:**
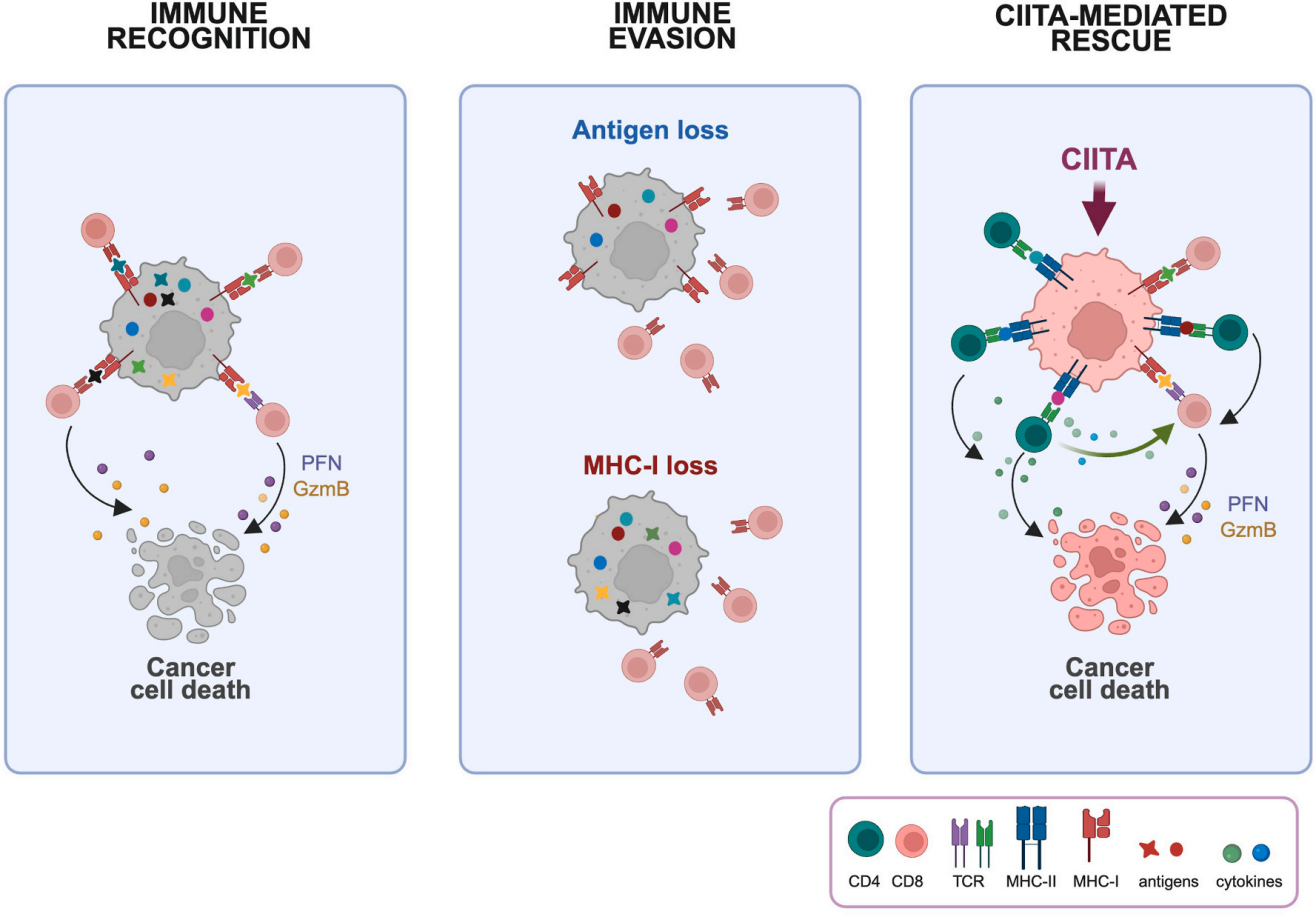
**Mechanistic model of tumor immune evasion through MHC or antigen loss and restoration of antigen presentation via CIITA.** Schematic representation of tumor immune escape mediated by loss or downregulation of tumor antigens and/or major histocompatibility complex (MHC) molecules, leading to impaired antigen presentation and reduced T-cell recognition. Expression of the class II transactivator (CIITA) restores antigen presentation pathways, enabling display of tumor-derived peptides and promoting CD4+T-cell-mediated immune recognition. Created in https://BioRender.com.

**Fig. 2:**
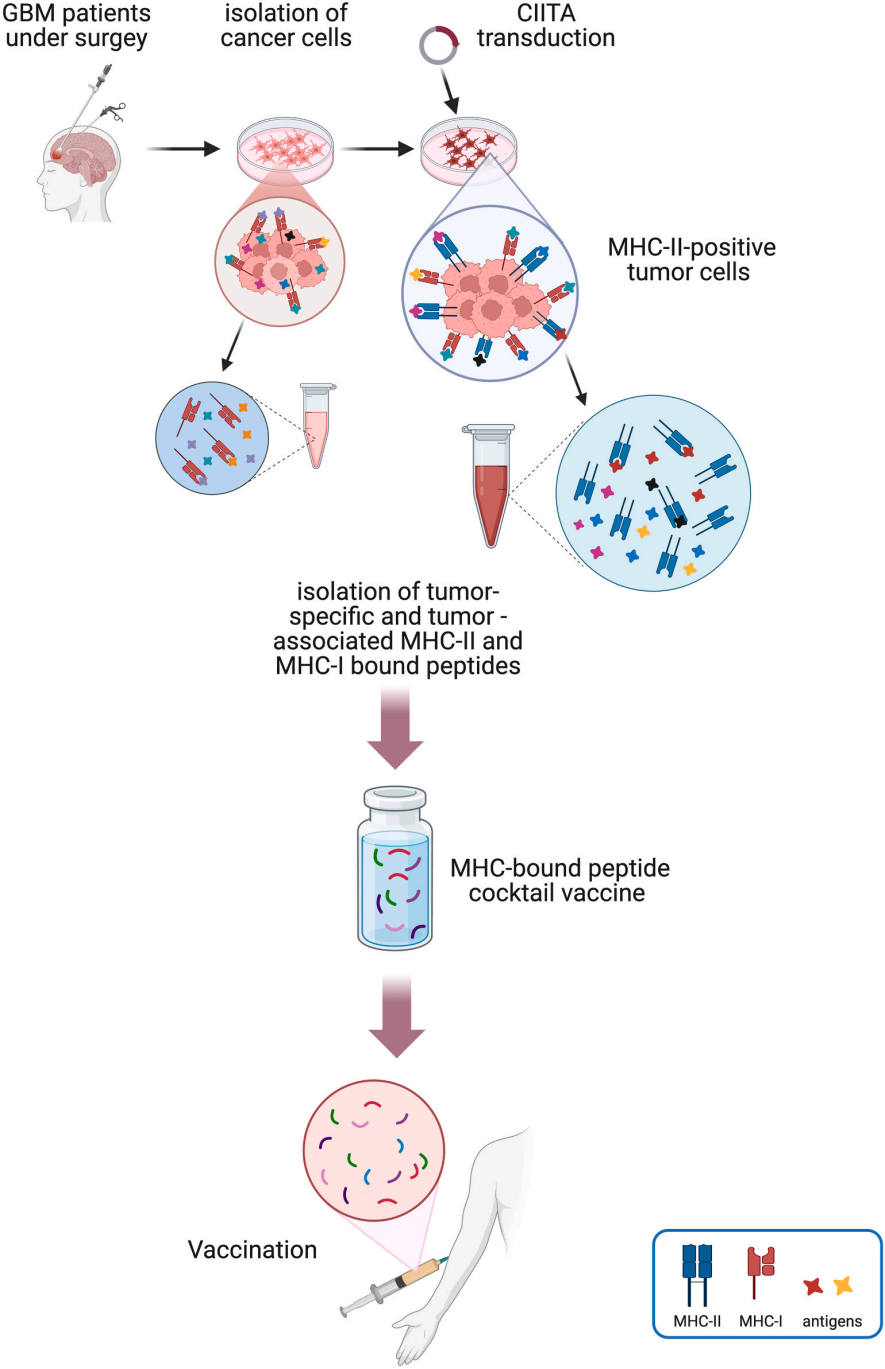
**GBM-CIITA cells expressing functional MHC class II molecules provide a novel source of MHC-II-restricted tumor peptides for anti-tumor vaccine development**. Patient-derived GBM cells are typically MHC–II–negative and show low MHC-I surface expression. CIITA transduction restores MHC-II expression, enabling presentation of previously hidden tumor-specific peptides. Peptides eluted from MHC-I and MHC-II complexes can be purified, selected for tumor specificity, and combined to generate therapeutic vaccine candidates for clinical testing in cancer patients. Created in https://BioRender.com.

Glioblastoma remains a formidable therapeutic challenge, primarily due to its immunosuppressive microenvironment, intratumoral heterogeneity, and impaired antigen presentation. The frequent downregulation of MHC-I and MHC-II limits effective CD8^+^ and CD4^+^ T cell responses, constraining the efficacy of immunotherapies, including peptide vaccines. CIITA-mediated restoration of MHC-II not only reprograms GBM cells into functional surrogate antigen-presenting cells but, importantly, enables the identification of a wide repertoire MHC–II–restricted immunogenic peptides [Fig. 1] [Table 3]. These peptides, particularly those shared across patients, provide a translationally feasible avenue for developing broadly applicable peptide vaccines [Fig. 2)] While peptide vaccines have demonstrated safety and immunogenicity, clinical efficacy has been modest due to antigen escape, MHC restriction, and immune suppression. In this context, CIITA-mediated strategies may represent a turning point. While ICIs have shown limited efficacy as monotherapies in GBM, increasing evidence indicates that their activity critically depends on the presence of a pre-existing tumour-specific T-cell response. We therefore hypothesise that CIITA-driven MHC–II–restricted peptide vaccination can provide the critical immune priming required to convert ICI-refractory GBM into ICI-responsive tumors, thereby overcoming a major barrier to the clinical success of immune checkpoint inhibition in this disease. A similar rationale may apply to combination immunotherapy involving MHC–II–restricted peptide vaccination and radiotherapy as the latter procedure has been shown to release potentially relevant tumor antigens following tumor cell necrosis that could sensitize tumor-specific T cells which then could be further rescued by peptide vaccination.

**Table 3:** Comparison of vaccine strategies in GBM.

Strategy	Strengths	Limitations	Translational impact
Peptide vaccines	- Simple, safe, well tolerated - Can be personalized (HLA-matched, neoantigens) - Proven immunogenicity	- Limited by HLA restriction - Antigen escape & tumor heterogeneity - Mostly modest clinical efficacy	Useful as backbone for personalized vaccines; need improved antigen selection and combination with checkpoint blockade
Dendritic cell vaccines	- DCs are potent APCs - Can present multiple antigens simultaneously - Induce both CD4^+^ and CD8^+^ T cell responses	- Labor-intensive, costly manufacturing - Limited efficacy in trials (e.g. DCVax-L, ICT-107) - Methodological concerns (external controls, selection bias)	Proof of concept for *ex vivo* antigen loading, but limited scalability and unclear clinical benefit
CIITA-based strategies	- Restore MHC-II expression on GBM cells - Allow direct identification of naturally presented HLA-II ligands - Prime CD4^+^ T cells, sustaining CTL activity - CIITA exerts intrinsic tumor-suppressive effects- Can be combined with viral/nanoparticle delivery or oncolytic viruses (e.g. HSV/T-VEC)	- Still preclinical/early translational stage - Requires optimization of delivery systems and safety validation	Provides a next-generation platform: identifies shared, physiologically relevant peptides for vaccine design and reprograms tumor immunogenicity

**Fig. 3:**
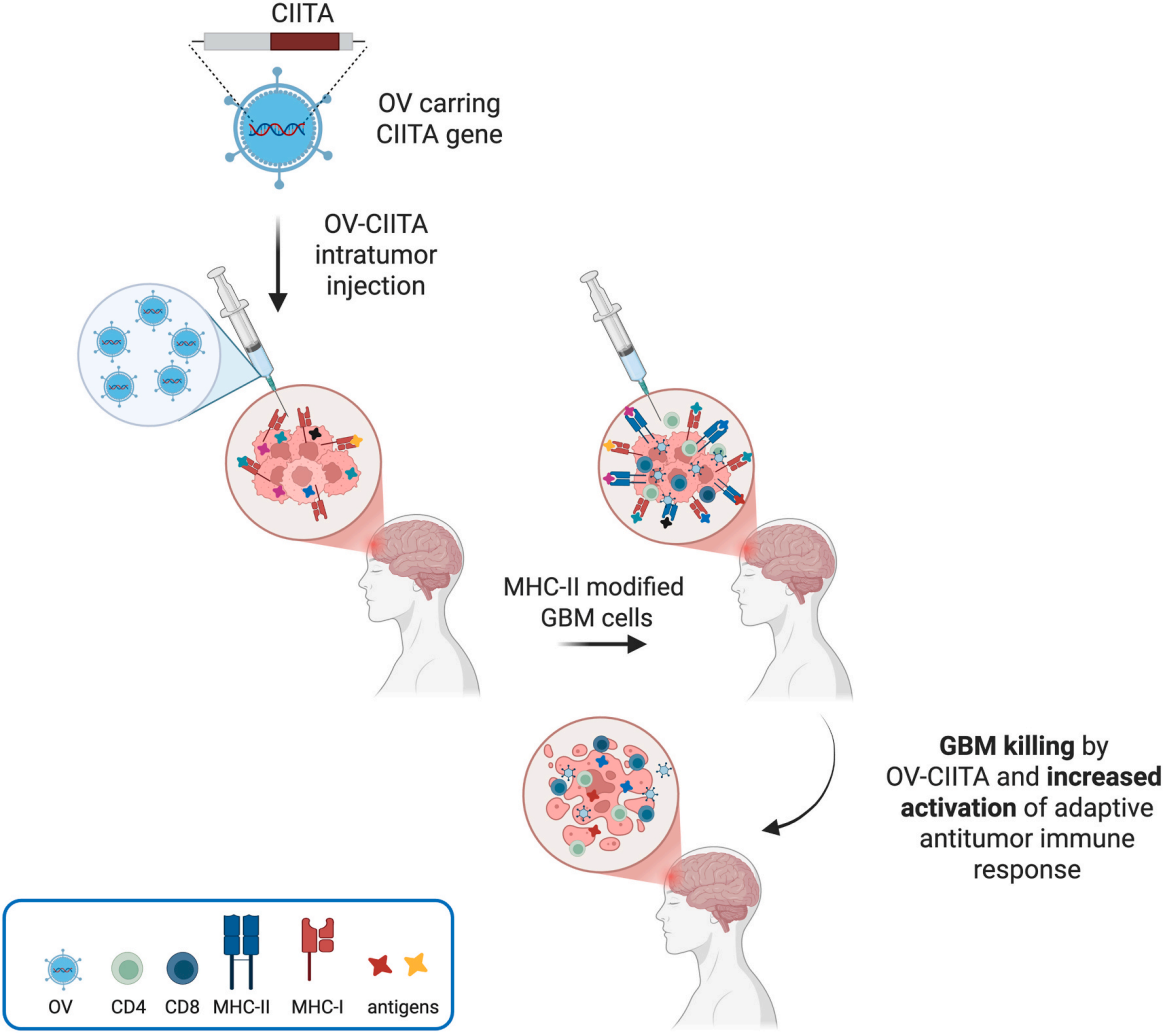
**Treatment of GBM with OV-CIITA constructs can amplify anti-tumor recognition and cell death.** Intratumoral injection of an oncolytic virus carrying the CIITA gene (OV-CIITA) induces MHC-II expression in GBM cells, promoting tumor antigen presentation and activation of CD4^+^ and CD8^+^ T cells, leading to enhanced antitumor immunity and GBM cell killing. Created in https://BioRender.com.

Recently, it has become apparent that oncolytic virotherapy may have an important application also in the contest of GBM (reviewed in Rahman and McFadden, 2021) [80] in which the oncolytic action is coupled with an increase of immune activation [81] Indeed, we have recently demonstrated in an orthotopic animal model of GBM that the intratumoral administration of an Herpes Simplex (HSV-1) oncolytic virus results not only in rejection or strong retardation in tumor growth but also, and importantly, in the acquisition of protective anti-tumor memory response capable to counteract a second challenge of the tumor [82]. Thus, inserting CIITA into an oncolytic viral vector such as HSV-1 may result in an optimal tool to increase even more the immune response to GBM by coupling the oncolytic effect with an optimal presentation of relevant immunogenic tumor antigens [Fig. 3]. Integrated approaches taking advantage of the acquired knowledge of using CIITA as an immune response inducer and/or amplifier of adaptive immunity provides a roadmap for overcoming current limitations in GBM immunotherapy, bridging mechanistic insight with actionable therapeutic strategies.

## Authors’ contributions

**M. Shallak**: writing-review and editing; **A.K.B Shaik**: writing review and editing **A. Gatta:** editing; **M. Volpi**: editing; **R.S. Accolla:** Conceptualization, writing-original draft, writing-review and editing; **G. Forlani:** Conceptualization, Resources, writing-original draft, writing-review and editing. All authors read and approved the final version of the manuscript.

## Conflict of interest statement

All authors have read the journal's policy on disclosure of potential conflicts of interest and all authors declare that don't have any financial or personal relationship with organizations that could potentially be perceived as influencing the described research.
